# *TGF-β* polymorphism and its expression correlated with CXCR4 expression in human breast cancer

**DOI:** 10.1186/1753-6561-7-S2-P38

**Published:** 2013-04-04

**Authors:** Julie Massayo Maeda Oda, Karen Brajão de Oliveira, Roberta Losi Guembarovski, Alda Losi Guembarovski, Glauco Akelinghton Freire Vitiello, Patricia Midori Murobushi Ozawa, Bruna Karina Banin Hirata, Vânia Darc de Castro, Maria Angelica Ehara Watanabe

**Affiliations:** 1 Department of Pathological Sciences, Biological Sciences Center, State University of Londrina, Londrina, PR, Brazil; 2 Laboratory of Pathology, Healthy Sciences Center, State University of Londrina and Laboratory of Applied Pathology, MICROPAR, Londrina, PR, Brazil

## Background

It is known that the transforming growth factor beta (TGF-β) can act as both a tumor suppressor and as a significant stimulator of tumor progression, invasion, and metastasis. It has been suggested a link between TGF-β and CXCR4 expression in human breast cancer cells, which may be one of the mechanisms of TGF-β mediated enhancement of metastatic potential in breast cancer cells. Therefore, the objective of the present study was to investigate the TGF-β T869C polymorphism and its expression correlated with CXCR4 expression in breast cancer patients.

## Patients and methods

Genomic DNA was obtained from 21 samples of peripheral blood or from normal tissue previously fixed in formalin and embedded in paraffin for TGF-β T869C polymorphism analyses. Total cellular RNA was extracted from the same 21 patients, but from fresh tissue (tumor and adjacent healthy from the same breast) to expression analysis by Real Time PCR.

## Results

No significant differences were observed in genotype distribution according to clinic pathological characteristics. TGF-β mRNA expression was assessed according to T869C polymorphism and CC patients presented a higher TGF-β expression but not significant when compared to other genotypes (p=0.064). A positive correlation was observed in relative mRNA expressions of CXCR4 and TGF-β (p= 0.020). It is known that overexpression of TGF-β by both tumor and stromal tissue can facilitate the development of metastases, mainly by TGF-β stimulated angiogenesis and increased tumor cell motility.

## Conclusion

Our findings suggested a role of these genes as progression markers for breast carcinoma.

## Financial support

CNPq, CAPES, Fundação Araucaria and PROPPG-UEL.

